# Plant–Microbe Interactions in Sorghum Nutrient Acquisition: Roles of Rhizosphere Microbes and N, P, and K Transporters

**DOI:** 10.3390/plants15152273

**Published:** 2026-07-24

**Authors:** Prantika Datta, Rozalynne Samira

**Affiliations:** Department of Plant and Soil Science, Texas Tech University, Lubbock, TX 79409, USA

**Keywords:** *Sorghum bicolor*, root microbiome, transporters, arbuscular mycorrhizal fungi, sustainable agriculture

## Abstract

*Sorghum bicolor* is an important cereal crop because of its tolerance to drought, high temperature, and low input requirements. However, the low productivity of sorghum is still due to the inadequate nutrient status and inappropriate use of nitrogen (N), phosphorus (P), and potassium (K). These nutrients are essential for plant growth, metabolism, abiotic stress tolerance, and yield development. Nutrient uptake in sorghum is regulated by the plant, the rhizosphere microbiome, and close interactions between the root system and membrane-localized transporter systems. This review summarizes the molecular interaction between the root and microbes that control the N, P, and K transporter genes in sorghum. It initially describes the architecture of sorghum roots, root exudation, and the key elements that govern rhizosphere microbiome assembly. It goes on to explain the molecular structure of significant nutrient transport systems, such as nitrate, ammonium, phosphate, and potassium transporters. Other important microbial groups related to nutrient uptake identified include diazotrophs, phosphate-solubilizing, potassium-mobilizing, and arbuscular mycorrhizal fungi. The focus is specifically on the interaction of plants and microbes that affect transporter gene expression and symbiotic acquisition of nutrients. In general, the review highlights an integrated framework of the interaction of root characteristics, microbial communities, and transporter networks in the process of controlling nutrient uptake in sorghum, and it determines valuable directions for future studies and crop enhancement.

## 1. Introduction

**Figure 1:**
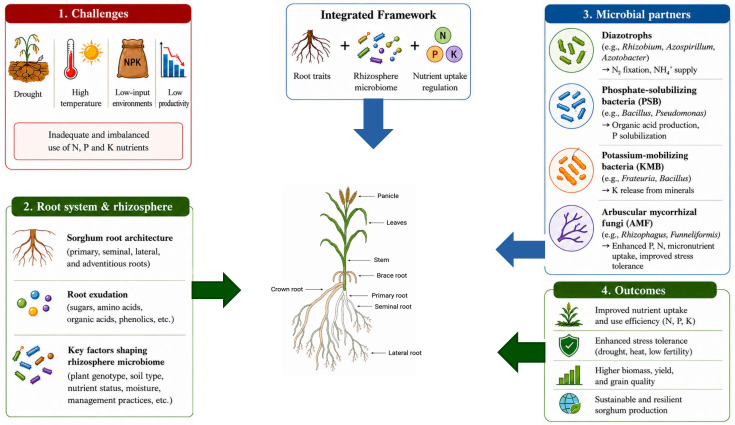
Integrated Framework of Root–Microbe Interactions Regulating N, P, and K Uptake in *Sorghum bicolor*. Schematic representation of sorghum root architecture, highlighting the major root classes (brace root, crown root, primary root, seminal root, and lateral roots) together with the aboveground shoot structures whose development underlies the root traits. Sorghum root traits and rhizosphere microbial communities interact to enhance nitrogen, phosphorus, and potassium acquisition through biological fixation, nutrient solubilization, mobilization, and uptake regulation, resulting in improved nutrient-use efficiency, stress tolerance, productivity, and sustainable crop production. Root traits are the upstream, foundational layer of this framework because they physically and chemically determine which microbial partners are recruited; the microbiome and transporter layers shown here are therefore consequences of root-level regulation rather than independent inputs, consistent with the unifying hypothesis stated in the Introduction.

*Sorghum bicolor* is a world-important cereal crop that is adapted to drought, heat, and low inputs; its growth is limited by insufficient amounts of irrigation water, low soil fertility, and minimal external inputs [1,2]. Essential nutrients are required for a plant to complete its life cycle and are known as macronutrients (C, H, O, N, P, K, Ca, Mg, S) and micronutrients (Fe, Mn, Zn, Cu, B, Mo, Cl, Ni) [3,4]. Among these, nitrogen (N), phosphorus (P), and potassium (K) are the most important macronutrients needed in the highest quantity and are the most likely to limit plant growth and yield. Fertilizers account for 40–60% of productivity in many cropping systems [5], but nutrient-use efficiency remains low, estimated at 33%, 16%, and 19% for N, P, and K, respectively, for global cereal production [6]; with much going to the atmosphere as greenhouse gases or to the environment as eutrophication forms of the nutrients [7]. Therefore, enhancing the acquisition and use efficiency of N, P and K in sorghum is key for yield stability and environmental sustainability. These nutrients cause characteristic physiological reactions in sorghum when they are deficient. The effects of N deficiency include chlorosis, growth inhibition, and changes in root-to-shoot ratio, and N deficiency also resulted in similar responses as combined N-P-K deficiency at the plant level [8]; and at the metabolic level, N starvation resulted in a decrease in the synthesis of N-containing macromolecules and in an increase in protein degradation and phenylpropanoid biosynthesis [9,10,11]. Phosphate deficiency induces root growth in sorghum and rice but not in Arabidopsis and induces anthocyanin accumulation and production of phosphatases. The low levels of potassium lead to decreased photosynthesis and root growth and disrupt ionic homeostasis in the cells, triggering the activation of signaling pathways involving reactive oxygen and ethylene [12,13,14,15,16]. The uptake of nutrients in sorghum is largely regulated by the root system—architecture, branching, root hairs, and exudation—as well as the ability to acquire them, which is strongly influenced by the rhizosphere microbiome and nutrient availability in the soil, and so nutrient acquisition is best seen as a plant–microbe process [17,18,19,20]. The root traits (Figure 1) influence the microbes that are recruited to the rhizosphere; the microbes in turn increase nutrient availability through biological fixation, mineral solubilization, release of organic acids, production of hormones, and by making the nutrients accessible for uptake, which is regulated by membrane-localized transporter systems, including NO_3_^−^/NH_4_^+^ transporters for nitrogen, high-affinity transporters for phosphorus, and transporter systems and channels for potassium [21,22]. The beneficial microbes, such as arbuscular mycorrhizal fungi, *Rhizophagus irregularis*, *Funneliformis mosseae*, and plant-growth-promoting rhizobacteria, Azospirillum, Bacillus, and Pseudomonas, directly regulate the activity of the transporters and nutrient signaling most significantly by inducing the expression of transporters during AMF symbiosis [23]. This review highlights the molecular mechanisms and the interactions between the root and rhizosphere microbiome, like assembly, architecture of transporters, microbial groups, and the role of plant–microbe coordination on nutrient-use efficiency.

The review is built upon this single unifying hypothesis that under low-input cultivation, sorghum’s nutrient use efficiency and adaptability to stress are a function of the coordinated regulation of root system architecture, root exudate signaling, N-P-K transporter gene networks, and the assembly of a beneficial rhizosphere microbiome, not just one or two of these components. Coordinated root-microbial-transporter networks (Section 2, Section 3 and Section 4) lead to resilience mechanisms (Section 5), which are mediated by the phenotypes of the roots (Section 2) and their recruitment of microbial partners (Section 4).

## 2. Sorghum Root System and Rhizosphere Microbiome Assembly

### 2.1. Root System Architecture

Sorghum has a stress-adaptive root system that is efficient in water and nutrient uptake and in maintaining soil microbial communities. All roots have a function for anchorage and uptake of water and nutrients from the soil [24]. The architecture of the root system changes over time and depends on the type of root system and the rate at which it grows into the soil. The seminal roots on which the germination of sorghum occurs give rise to the root system, and the nodal, crown, or adventitious roots, which grow subsequently on the shoot nodes (Figure 1). Seminal roots are important for early water and nutrient uptake and for seedling growth, while nodal roots become more important at later growth stages [25]. The growth rate of the sorghum root structure in soil is 2–3 cm/day [25]. These architectural and developmental capabilities are significant since they dictate the depth of soil exploration, the angle of the root, and the direction and degree of branching, as well as the contact of the root with the microbes of the rhizosphere. Sorghum possesses a deeper, less dense, stress-tolerant root system, and this could be the reason for its high performance under drought and other abiotic stresses [26]. Maize and sorghum can vary significantly in these architectural traits, as sorghum has only one seminal (primary) root, while the coleoptile-borne nodal roots appear at the fourth to fifth leaf stage compared to maize, which has 3–7 seminal and scutellar roots and the nodal roots emerge at the second leaf stage, with a slower early root trajectory that is also less-branched than maize [25], and consistent with sorghum’s typically deep, but less dense mature root system. This is also a genetically determinate variation; a GWAS/QTL study of root-angle traits across 428 sorghum germplasm from the Ethiopian breeding program identified markers associated with grain-yield performance for water-limited environments [26], suggesting that GWAS/QTL analysis can be used to dissect heritable variation in root-architecture, which to date has been defined in terms of drought adaptation but is likely to apply to nutrient-foraging traits under the low-input conditions considered in this review.

### 2.2. Rhizosphere Microbiome Assembly

Interactions between plants and microorganisms in the rhizosphere are largely controlled by biochemical signals, and this is now widely recognized as a common feature of the rhizosphere environment. Plant–microbe interactions in this zone can be understood in two ways. First, there are many factors influencing the microbiota of the rhizosphere. These include abiotic factors such as root exudates, soil type, climate, and biotic factors such as diseases, insect pests, plant species, genotype, root traits, and plant growth stage. Second, rhizosphere microbes are also able to affect plant growth and disease resistance through available mineral nutrients and phytohormone production. These beneficial microbes are known as plant growth-beneficial rhizosphere microorganisms (PGBR) [27]. Under low phosphorus conditions, the reference genotype BTx623 of sorghum shows significant modifications of root system architecture with longer roots, larger root surface, and lateral root development. The changes reflect sorghum’s response to a P-deficient environment and its increased root system to search out more soil volume and higher opportunity to access available P [28]. Besides this, QTL analysis in a low-P environment revealed that the external area of the root and other root morphology characteristics are strongly associated with phosphorus uptake efficiency and eventually grain yield in sorghum [29]. Recent studies on the nodal root bud development revealed that there is an upregulation of *WOX4*, *LAX2*, *PIN4*, *WOX11*, *WOX5*, and *PLT* genes during the root bud initiation and outgrowth [30]. These genes are associated with meristem, hormone signaling, development of root caps, and cell proliferation [31]. This implies that sorghum root architecture is not just a physical property, but it is constructed using a controlled developmental program, which subsequently determines nutrient uptake and microbial colonization [32]. Root exudation is a significant way in which sorghum affects its rhizosphere microbiome. One of the most important compounds is sorgoleone, a specialized substance released from sorghum root hairs. It can change the structure of bacterial and archaeal communities in the rhizosphere, slow the early development of microbial networks, and may influence nitrogen cycling, including nitrification [33]. For sorghum, the relative abundance of specific bacterial taxa was altered by root exudate (sorgoleone) addition to soil, and networks of co-occurrence among rhizosphere bacterial taxa were also delayed, showing an effect of this sorghum-specific metabolite on the assembly of bacterial communities [34]. This delay due to network formation by the rhizosphere bacteria is a plausible pathway for sorghum root exudation to influence rhizosphere nitrogen availability, and hence nitrogen-use efficiency, compared to other cereals; this is discussed further as a unique aspect of sorghum compared to other cereals in Section 4. In general, the assembly of microbiomes of the rhizosphere of sorghum can be considered a multilevel process. Morphological features of roots (root angle, branching, fine roots production, and root hairs) influence the physical template of microbe recruitment. The chemical environment of the rhizosphere is influenced by physiological traits such as responses to nutrient stress, distribution of carbon, and exudation. Developmental genes, hormone-signaling pathways, and phosphorus-starvation regulators, along with genotype-specific loci, play a role in the regulation of sorghum root architecture. These molecular regulators affect root angle, branching, production of fine roots, and exudation. All of these characteristics have the potential to influence the rhizosphere environment and could influence which beneficial microorganisms are attracted to the root surface. The same processes likely act upstream in the pathway that the positive root associates with microbes, regulating the expression and activity of the N, P, and K transporter genes in sorghum.

## 3. Molecular Architecture of N, P, and K Transporter Systems in Sorghum Bicolor

In sorghum, nitrogen (N), phosphorus (P), and potassium (K) are taken up and transported across specialized transporter systems located in the membranes of epidermal, cortical, endodermal, vascular, and intracellular cells [22]. Like other cereals, multigene transporter families are employed in sorghum, which enables adaptation of nutrient uptake to external supply, developmental stage, tissue type, and microbial interactions.

### 3.1. Nitrogen Transporters

**Figure 2:**
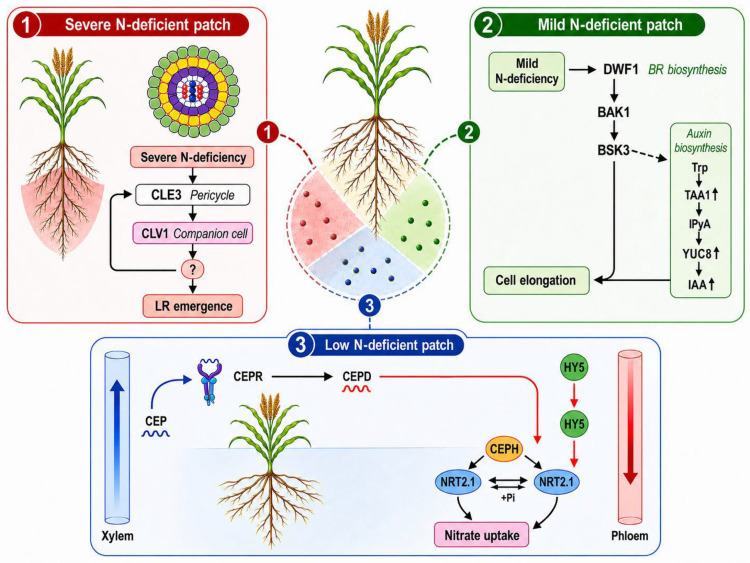
Molecular responses to severe, mild, and low nitrogen deficiency patches- redrawn from [22]. Severe N deficiency induces CLE3–CLV1 signaling to restrict lateral root emergence, whereas mild N deficiency activates brassinosteroid–auxin pathways that promote cell elongation and root foraging. Under patchy low-N conditions, CEP peptides produced in N-deficient roots move to the shoot and trigger CEPD- and HY5-mediated shoot-to-root signaling, which enhances *NRT2.1* expression and nitrate uptake in root zones where nitrate is available. Of these three responses, the CEP-CEPD-HY5-NRT2.1 shoot-to-root relay is the principal route by which sorghum matches nitrate uptake to patchy soil availability, while the CLE3-CLV1 and brassinosteroid-auxin pathways act mainly to set the boundary conditions (respectively restricting and permitting lateral root growth) within which that relay operates.

Nitrogen is an extremely dynamic nutrient in soil because it is unevenly distributed and may easily be converted from one chemical form to another through microbial- and chemical-driven reactions. N is highly heterogeneous in soil; nitrate and ammonium are often present as patches of high and low concentrations of nutrients around localized microsites. Microsites are small areas within the rhizosphere where nitrogen availability is different from the bulk soil due to the position, application, or mineralization of fertilizers, microbial nitrification, leaching, or site-specific moisture conditions. Nitrogen deficiency causes a decrease in leaf area, chlorophyll content, photosynthetic rate, and biomass accumulation in sorghum [35]. Plants mostly have nitrogen in the form of nitrate (NO_3_^−^) and ammonium (NH_4_^+^). Both are transported into root cells via different transporter families, such as *NRT1*, *NRT2*, and *AMT* transporters. In particular, *NRT1.1/NPF6.3* is crucial, as it not only acts as a nitrate transporter but is also a nitrate sensor [36]. In sorghum, ammonium transporter genes have been identified, and *SbAMT3;1* and *SbAMT4* are locally induced in roots colonized by arbuscular mycorrhizal fungi, especially in cortical cells containing arbuscules or neighboring colonized cells, suggesting a role in mycorrhiza-associated ammonium transport [37]. When N is extremely scarce in a localized root zone, plants limit the emergence or elongation of lateral roots. This response allows carbon and energy to be redirected toward root zones or plant organs. Severe N limitation induces expression of the *CLE3* gene in root pericycle cells. *CLE3* belongs to the CLAVATA3/Embryo surrounding region-related family of small secreted signaling peptides. The *CLE3* signal is then received by the CLAVATA1 (*CLV1*)-related signaling pathway in the companion cells of the phloem tissue, which inhibits the emergence of lateral roots. When soil N is low in the vicinity of the plant, it turns off unnecessary lateral root growth and focuses energy on more productive areas of the root system [38]. Under mild N deficiency, the reaction is quite different. In such cases there is still some nitrogen in the soil; thus, the plant does not prevent root exploration but encourages it. In response to mild N limitations, brassinosteroid-related signaling pathways are activated, such as genes involved in BR biosynthesis (e.g., *DWARF1*, *DWF1*) and genes coding for components in BR signaling (e.g., BRI1-associated receptor kinase 1, *BAK1*; Brassinosteroid Signaling Kinase 3, *BSK3*). These BR signals are found to interact with auxin biosynthesis and transport pathways such as *Tryptophan Aminotransferase 1* (*TAA1*), *YUC* genes, and Indole-3-acetic acid (IAA) related responses. These hormonal pathways work together to elongate cells and to stimulate lateral root development. This helps the root system to grow deeper into the ground and enhances the likelihood of taking up nitrate in the microsites. This root-foraging response is significant as it enables the plant to match nutrient needs with the plant’s energetic costs of root growth [39,40]. In the low-N patch model (shown in Figure 2), *CEP* genes are induced. *CEP* is a family of small, secreted peptide hormones that are produced from short precursor proteins, which then undergo processing to produce mature *CEP* peptides that can translocate from N-deficient root regions to the shoot through the xylem. CEP peptides are received in the shoot by the CEP receptor proteins, which trigger the mobilization of shoot-derived signals. Of these downstream signals, CEPD are small polypeptides that are translatable between the leaves and move back to root regions containing nitrate, where they stimulate the expression of the high-affinity nitrate transporter gene *NRT2.1*. Another mobile regulatory protein is HY5, a bZIP transcription factor that can translocate from the shoot to the roots and stimulate nitrate uptake at least by regulating *NRT2.1* expression. These signals enable the plant to adapt its internal demand for nitrogen to external nitrate supply, such that roots in the microsite with nitrate uptake capacity grow more abundantly [41,42,43,44]. This nitrogen uptake system may also be able to interact with beneficial bacteria. Arbuscular mycorrhizal fungi (AMF) can increase the surface area of the roots that can acquire nitrogen and the quantity of the N pools that might not be readily available to the roots. AMF hyphae had access to an organic matter patch, where they grew in that patch and transferred significant amounts of N to the host plant, leading to increased plant N and enhanced biomass. In sorghum, this could be through the action of the sorghum ammonium transporters, *SbAMT3;1* and *SbAMT4*, which are locally induced in mycorrhizal roots, especially in the cortex surrounding arbuscule-containing cells [45]. Thus, AMF uptake of N from soil is a coordinated process that includes fungal uptake, internal fungal transport, externalization of ammonium in the vicinity of the plant–fungus interface, and plant uptake via sorghum ammonium transporters. Thus, nitrogen acquisition is a process that is integrated with transporters, peptide signals, hormone pathways, root architecture, and microbial associations. This coordination can be especially relevant in sorghum in low-input, stressful conditions to increase nitrogen-use efficiency.

### 3.2. Phosphate Transporters

**Figure 3:**
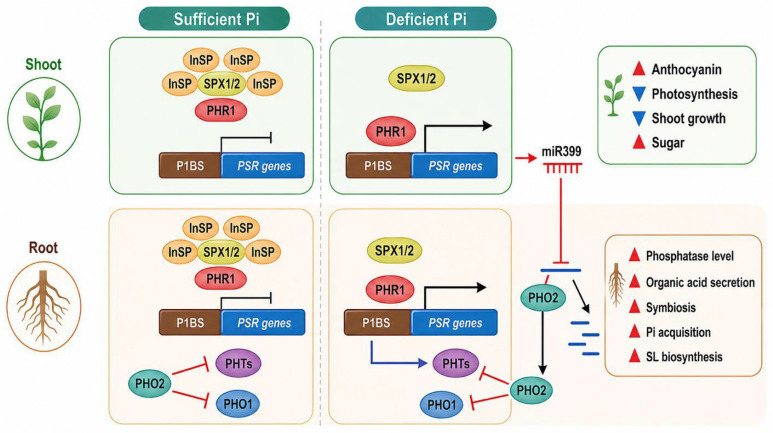
Phosphate starvation signaling and adaptive responses under sufficient and deficient Pi conditions; redrawn from [22]. Under sufficient Pi, SPX1/2 interacts with PHR1 and suppresses phosphate starvation response (*PSR*) genes in both shoot and root. Under deficient Pi, this repression is relieved, allowing PHR1 to activate *PSR* genes. In the shoot, Pi deficiency induces *miR399*, which suppresses PHO2-mediated regulation. In the root, reduced PHO2 activity enhances the accumulation or activity of PHTs and PHO1, promoting Pi uptake and transport.

Phosphorus plays a crucial role in the transfer of energy, nucleic acid synthesis, membrane structure, and various metabolic reactions. Total soil phosphorus can be relatively abundant; however, the concentration of plant-available inorganic phosphate (Pi) in the soil solution is often low because much of the soil P is immobilized by adsorption, precipitation, or association with organic and mineral fractions. Therefore, Phosphorus is primarily taken up in plants as inorganic phosphate (Pi) and is primarily translocated into the plant by *PHT1* family phosphate transporters in root cells [46,47]. (Figure 3) illustrates that at a sufficient Pi level, the plant maintains the level of phosphate starvation response at a constant level. In this state, SPX proteins are phosphate-status sensors that can interact with the Phosphate Starvation Response 1 (PHR1), a large transcription factor controlling the genes of phosphate starvation response. In cases where internal Pi is sufficient, the SPX–PHR1 interaction inhibits PHR1 from activating superfluous Pi-starvation genes. This is significant since phosphate scavenging, secretion of organic acids, root remodeling, and the induction of transporters all use energy. Thus, in the conditions of Pi-sufficiency, the plant can preserve phosphate homeostasis and does not over-activate stress reactions [48,49]. This repression is emitted under Pi deficiency. PHR1 is activated and triggers a wide range of genes in the phosphate starvation response. When Pi is low, *miR399* is highly induced and translocated via the phloem to control phosphate homeostasis. Its primary target is PHO2, a ubiquitin-conjugating enzyme that is an inhibitory regulator of phosphate transport. In the presence of *miR399* that suppresses Phosphate 2 (PHO2), the phosphate transporters and other phosphate-loading proteins like Phosphate Transporters (PHTs) and PHO1 are either more active or more stable. This enhances Pi uptake by roots and facilitates Pi translocation by roots to shoots [49,50,51]. It is also indicated in the figure that Pi deficit not only affects the root but also the entire plant. Pi limitation usually decreases photosynthesis and shoot growth and enhances sugar buildup and anthocyanin synthesis in shoots. Pi starvation can promote organic acid secretion, alter root architecture, augment Pi uptake ability, and stimulate strigolactone production in roots. These modifications contribute to the fact that the plant can mobilize the phosphate in the rhizosphere, as well as enhance its capacity to survive in low-P environments. *PHT1* transporters are likely to be significant in Pi uptake in the soil and redistribution of Pi in the plant in the case of cereals like sorghum. Nevertheless, the action of such transporters is not determined by the abundance of transporters but by the greater part of the SPX–PHR1–miR399–PHO2 regulatory network [51,52]. This phosphate signaling pathway has a strong connection with plant–microbe interactions as well. Arbuscular mycorrhizal fungi and phosphate-solubilizing bacteria can augment the availability of Pi around the root by expanding the zone of absorption, discharging acidic substances, or mobilizing fixed phosphate. But this additional phosphate can only be utilized by the plant when the system of its transporters is functioning and is controlled in a proper way. The uptake of phosphates can then be viewed as a collaboration between the mobilization at the soil level and signaling at the plant level. Microorganisms can boost the supply of available phosphate, and the plant can use the PHT transporters and phosphate starvation responses to efficiently absorb and distribute the available phosphate [49,53].

### 3.3. Potassium Transporters

**Figure 4:**
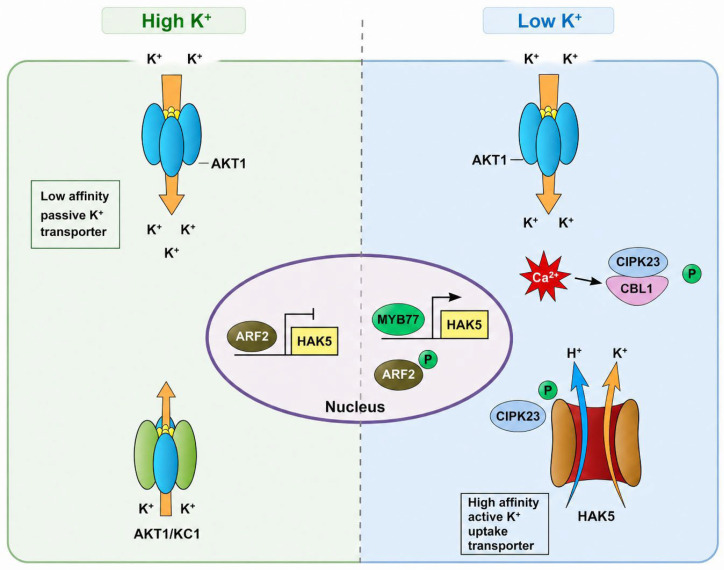
Regulation of potassium uptake under high and low K^+^ conditions- redrawn from [22]. Under high K^+^ conditions, K^+^ mainly enters root cells through low-affinity passive transporters such as AKT1, while high-affinity HAK5 expression remains suppressed by ARF2. Under low K^+^ conditions, calcium signaling activates the CBL1–CIPK23 pathway, which promotes AKT1 and HAK5 activity. At the same time, MYB77 enhances HAK5 expression, supporting high-affinity K^+^ uptake to maintain potassium balance in roots. The CBL1/CBL9-CIPK23 calcium-sensing module is the primary switch governing this transition, since it directly activates both AKT1 and HAK5, whereas the ARF2-MYB77 transcriptional module fine-tunes the magnitude of HAK5 expression once the switch has been triggered.

The most common inorganic cation in the plant cell is potassium, which is critically important in osmotic regulation, stomatal movement, activation of enzymes, membrane potential, and drought and salinity tolerance. In contrast to nitrogen and phosphorus, potassium is not used in the formation of organic molecules, and it plays a vital role in the ionic balance of the cell and physiological activity. (Figure 4) shows that plants predominantly use low-affinity uptake systems in situations where soil K^+^ is adequate. Under this situation, K^+^ enters the root cells through inward-rectifying K^+^ Transporter 1 (AKT1). When K^+^ is readily available, the plant relies mainly on low-affinity uptake systems. Thus, the expression of the High Affinity K^+^ transporter 5 (HAK5) remains low. Auxin Response Factor 2 (ARF2) is one such regulator in this state that is a transcriptional suppressor of HAK5 when the conditions are K-sufficient. This maintains the high-affinity uptake pathway in an inactive state when not required and assists the plant in conserving energy [54]. When external K is limited, the plant alters its strategy. Reduced availability of K^+^ causes calcium signaling in root cells. Calcineurin B-Like Protein 1 (CBL1) and Calcineurin B-Like Protein 9 (CBL9) are the calcium sensors that activate CBL-Interacting Protein Kinase 23 (CIPK23). CBL-CIPK23 complex then phosphorylates and activates the major K^+^ uptake components, such as AKT1 and HAK5. This signaling pathway allows the plant to enhance its ability to take up K^+^ quickly in response to deficient conditions. The importance of HAK5 increases since it is a high-affinity K^+^ transporter and can act at a very low external K concentration [55,56]. There is both transcriptional and post-translational regulation of HAK5. The transcription factor protein with the MYB domain 77 (MYB77) enhances the expression of HAK5 under low K^+^ conditions. Meanwhile, ARF2 is phosphorylated, thus impairing its HAK5 promoter-suppressing capability. This frees HAK5 of transcriptional repression and permits increased K uptake. Following the production of HAK5, its activity can be boosted by CBL-CIPK signaling kinase. Recent studies also back the notion that HAK5 relies on the proton motive force to facilitate high-affinity K uptake, which is why it is particularly effective at very low K availability [55,57].

## 4. Plant–Microbe Interactions

### 4.1. Microbial Regulation of Nitrogen Uptake and Transporters

**Figure 5:**
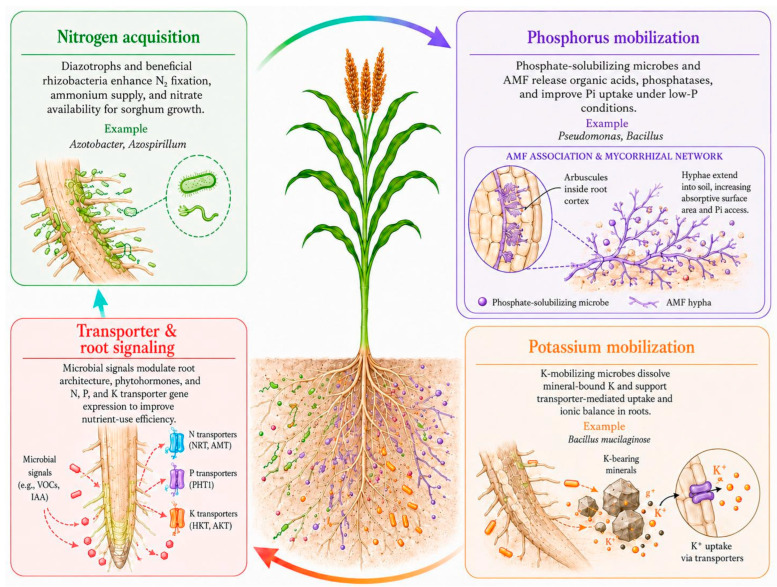
Schematic representation of microbial-mediated nutrient acquisition in sorghum. Beneficial microorganisms enhance nitrogen acquisition, phosphorus mobilization, potassium mobilization, and nutrient transporter signaling, resulting in improved nutrient uptake and plant growth. Among the pathways, diazotroph-mediated N fixation and AMF-mediated P/micronutrient transfer represent the two best-supported and quantitatively largest contributions to sorghum nutrient acquisition (Section 5.1, Section 5.2 and Section 5.3); phosphate- and potassium-solubilizing bacteria and endophyte-mediated stress tolerance (Section 5.4) act as secondary, context-dependent contributors whose effect sizes vary more with soil type and microbial strain.

In addition to providing nutrients in the soil, rhizosphere microbes directly influence the uptake machinery of the plant by inducing expression of transporter genes, by modifying root architecture, and by producing signals that the plant’s nutrient sensing pathways respond to; this section discusses that direct microbial regulation of N, P, and K uptake, which complements the acquisition mechanisms of individual microbial groups described in Section 5. The rhizosphere is a very narrow region of soil surrounding the roots of plants. It has various soil microorganisms, such as bacteria, fungi, protozoa, archaea, and viruses, that have a direct effect on the avenues of nitrogen [58]. Beneficial soil microbes help in providing nitrogen through organic matter mineralization, ammonification, nitrification, and biological nitrogen fixation (Figure 5). One of the most common plant-associated microorganisms is arbuscular mycorrhizal (AM) fungi. They penetrate the root cortical cells and enlarge the network of hyphae, which penetrate the soil beyond the root depletion zone [59]. The AM fungi can take up soil-borne nitrate and ammonium and transport the nitrogen to the host plant through the mycorrhizal uptake pathway [23]. In this pathway, the extraradical mycelium takes up nitrate (NO_3_^−^) and ammonium (NH_4_^+^) from the soil and incorporates nitrogen primarily into arginine, then carries it through the fungal hyphae to the intraradical mycelium within the root. Arginine is subsequently metabolized within the intraradical structures of fungi, and nitrogen released as ammonium is largely taken up by the plant’s ammonium transporters in the periarbuscular membrane of the root cortex cells [23,60]. One example of mycorrhizal control of the uptake of nitrogen is rice [61]. Rice plants infected by the arbuscular mycorrhizal fungus *Rhizophagus irregularis* that grow in low-nitrogen soils exhibited an increase in nitrate uptake via a mycorrhiza-specific mechanism. Only the cells in the root were induced to express the gene that encodes nitrate transporter OsNPF4.5 by arbusculus. This transporter was proven to be responsible for up to 40–42% of the total nitrate uptake under nitrogen-limited conditions [62]. In the case of suppression of this gene, nitrate uptake as well as the development of the arbuscule were lowered. The AM fungi selectively take up ammonium since it consumes less energy to be assimilated than nitrate. After uptake, the fungus assimilates this nitrogen into amino acids, especially arginine [63]. Arginine is an important compound to store and transport nitrogen in the fungal network. It is carried in the intraradical part of fungi, degraded, and nitrogen is released at the plant–fungi interface. In addition, there are several transporters of ammonium and nitrate that can transfer nitrogen to the plant and the fungus [23]. Many signaling molecules, such as phytohormones, small metabolites, and volatile organic compounds (VOCs), are produced by beneficial microorganisms. These signals can interact with root growth, control transporters, and interact with the nitrogen-sensing process [64]. Microbes induce the production of auxins that stimulate lateral root growth and development of the root hair to increase the root surface and uptake of nitrogen [65]. Cytokinins have been reported to regulate the root–shoot ratio and the amount of plant nitrogen distribution. Besides the direct impact on root gene expression, microbial VOCs may exert a systemic impact on the genes involved in N uptake/transport without contact with the root [66]. In the model plant species, *Arabidopsis thaliana*, inoculation of *B. subtilis* and *Phyllobacterium brassicacearum* resulted in significant induction of high-affinity nitrate transporters NRT2.1, NRT2.5, and NRT2.6, as well as ammonium transporters of the AMT1 family [67]. These changes were associated with the gain in shoot biomass and the total nitrogen content. This was also observed in wheat, as an inoculation with Plant Growth Promoting Rhizobacteria (PGPR) boosted nitrate uptake and nitrogen use efficiency at low nitrogen levels [68]. The beneficial microbes are also very effective in the assimilation of nitrogen. *Bradyrhizobium japonicum* increased the nitrogen fixation potential by increasing the activity of Nitrogenase and nitrate reductase (NR) and nitrite reductase (NiR) in the rhizosphere of soybean, making this crop more efficient in reducing nitrate. The impact of the microbes reduced the level of nitrate in tissues and improved the utilization of nitrogen as a whole [69]. *Pseudomonas* and *Azospirillum* enhance the amount of auxin in roots, which enhances the formation of lateral roots. This reaction is closely related to nitrate sensing via NRT1.1, which is linked to the availability of nitrates and auxin movement. The plant–microbe interactions integrate the uptake, transport, assimilation, and signaling of nitrogen into an orchestrated system [70]. Microbes have a simultaneous effect on the root architecture, expression of transporters, expression of enzymes, and signaling pathways. This combined control enables the plants to dynamically react to changes in the availability of nitrogen.

### 4.2. Microbial Regulation of Phosphorus Uptake and Transporters

Beneficial microorganisms also have an important role in phosphorus mobilization in addition to nitrogen acquisition. Most of the soil phosphorus is present in insoluble or fixed form, which does not directly benefit the plant, but is required as a macronutrient. Multiple phosphate-solubilizing bacteria and fungi can release the phosphate from these unavailable forms to soluble phosphate through the production of organic acids, protons, and extracellular enzymes. *Pseudomonas*, *Bacillus*, and *Rhizobium* are common genera that are phosphate-solubilizing [47]. Increased plant-available phosphate ions, such as HPO_4_^2−^ and H_2_PO^4−^, are released by chelation of the cations by organic acids such as gluconic and citric acid, and acidification of the rhizosphere [71]. Furthermore, organic P is mineralized by enzymes, such as phosphatases and phytases, which convert organic P compounds into orthophosphate, e.g., phytate [72,73]. Arbuscular mycorrhizal fungi also have additional effects on the uptake of phosphate, including the spreading of the hyphae beyond the limits of the root depletion zone, and the effect of stimulating phosphate transporters in plants (Figure 5). For instance, AMF caused upregulation of LePT4 (the tomato phosphate transporter) and increased phosphate uptake [74]. However, the combination of AMF and beneficial rhizobacteria could prove even more effective, as the fungus can help move P-solubilizing bacteria to sites of organic P in soil [75,76].

### 4.3. Microbial Regulation of Potassium Uptake and Transporters

A few soils have a high percentage of total K present as soluble or exchangeable K, but most soils have a high percentage of K bound in silicate minerals such as orthoclase, biotite, and mica. Potassium-mobilizing bacteria and fungi can convert this mineral-bound K into plant-available K^+^ mainly through organic acid production, proton release, acidolysis, chelation, and ion-exchange reactions [77,78]. As a result, microbial K mobilization increases the soluble K^+^ pool in the rhizosphere and improves the chance of K uptake by plant roots. Many other associations with fungi have been observed to improve plant K nutrition under K-limiting conditions. The ability of saprophytic fungus *Clitopilus hobsonii* to promote plant growth and increase K availability under K limitation was demonstrated in American sweetgum, for example, and is proposed to be mediated by K^+^ transporters of fungal origin [79,80]. Also, *Aspergillus aculeatus* increased the level of soluble K in perennial ryegrass and upregulated the *HAK8*, *HAK12*, and *HKT18* genes. The action of K-mobilizing microbes might be more effective when combined with K minerals such as feldspar, since the microbes can mobilize mineral sources, providing K for plant absorption [79]. Thus, the use of k-mobilizing microbes such as *Bacillus* sp., including *Bacillus mucilaginosus*, may be a beneficial biological tool to enhance the availability and ionic balance in plant roots (Figure 5).

### 4.4. Integrated Microbial Regulation of Nutrient Transporter Networks

Microbial signals and metabolites provide an important link between nutrient mobilization in the rhizosphere and nutrient uptake by plant roots. Microbes can produce phytohormones, volatile organic compounds, organic acids, siderophores, and other small metabolites that influence root growth, nutrient availability, and transporter activity. For example, microbial auxin production can stimulate lateral root formation and root hair development, which increases the root surface area and allows plants to explore a larger soil volume for nutrients [65]. At the same time, microbial organic acids and enzymes can increase the availability of fixed or organic nutrients, while fungal hyphae can extend the nutrient-absorbing zone beyond the root depletion area. These microbial activities work together with plant membrane-localized transporters, including NRT/NPF and AMT transporters for nitrogen, PHT transporters for phosphate, and AKT/HAK-type systems for potassium [22]. Therefore, plant-associated microbes not only supply or mobilize nutrients; they also support root development, nutrient signaling, and transporter-mediated uptake. In this way, nutrient acquisition becomes a coordinated plant–microbe process rather than a root-only function. However, the success of microbial inoculation depends on the plant genotype, microbial strain, soil nutrient status, and environmental conditions. A better understanding of these interactions will help develop sustainable nutrient management strategies that improve nutrient-use efficiency and reduce dependence on chemical fertilizers in crop production [53] (Table 1).

## 5. Role of Microbial Interactions in Sorghum Resilience

Sorghum is renowned for its drought and heat tolerance. Some sorghum varieties are known as poor hosts for root-knot nematodes, and a few resistance traits have been reported for downy mildew, anthracnose, and maize dwarf mosaic virus. The stress-resilience characteristics could be due to plant genetic modification, but they also appear to be modulated by metabolites secreted by the roots and the resulting rhizosphere microbial community. In particular, the roots of sorghum produce specific metabolites, like sorgoleone, that can affect the development of bacterial and archaeal communities in the rhizosphere and the growth of microbial networks. In recent years, evidence has also emerged that the soil microbiome has the potential to alter the metabolome of sorghum roots, which means microbial associations could play a role in sorghum defence and stress adaptation. The chemical compounds, microbial communities, and processes linking sorghum root chemistry to nematode, pathogen, and parasitic weed resistance, however, are largely unknown and need to be studied.

### 5.1. Plant-Growth-Promoting Microorganisms

A wide spectrum of bacteria and fungi are classified as Plant-Growth-Promoting Microorganisms (PGPMs) and are involved in plant growth promotion by contributing to nutrient solubilization, production of phytohormones, and abiotic stress mitigation. For example, *Azospirillum brasilense* is an associative diazotrophic plant-growth-promoting bacterium that can colonize cereal roots and promote plant growth through biological nitrogen fixation, phytohormone production, and stimulation of root development. In field-grown grain sorghum, inoculation with *A. brasilense* improved plant water status, increased total stover dry-matter yield, enhanced soil moisture extraction from deeper layers, and increased grain yield, suggesting that this bacterium can support sorghum performance under water-limited conditions [85,86]. Similarly, *Bacillus subtilis* improves the absorption of phosphorus by increasing the availability of phosphate compounds through the production of organic acids. Bacillus subtilis releases several gluconic acids, citric acid, and oxalic acid, which help to release insoluble phosphate compounds and enable the plant to absorb the free phosphate compounds [87]. It also acts to relieve plant stress by regulating hormones such as auxins and abscisic acid [88]. Apart from *B. subtilis*, other PGPR, like *Azospirillum*, *Pseudomonas*, *Burkholderia*, and endophytes like arbuscular mycorrhizal fungi (AMF) and *Penicillium*, can also enhance the availability of P and the tolerance to stress through different mechanisms such as root development support, hormone regulation, production of antioxidants, and hormone regulation [89]. New studies also indicate that PGPMs control the plant’s hormonal balance in times of stress. *Pseudomonas fluorescens* is known to enhance lateral root and root extension, which are important for root-water uptake [90]. Furthermore, these bacteria produce siderophores that chelate the Fe present in soil and enhance the amount of Fe made available to plants, which can help mitigate Fe deficiency in dry, alkaline soils [91]. When several PGPMs are combined in a mix and applied to sorghum, they are reported to have synergistic effects, by which more biomass is accumulated and even better yields under nutrient deficiency and water scarcity conditions than in the absence of them [92]. This is a microbial remedy that could help to reduce reliance on chemicals and enhance sustainable farming methods.

### 5.2. Phosphate-Solubilizing Microorganisms

In sorghum, the phosphate-solubilizing microorganisms (PSMs) increase the availability of plant phosphorus, which is the effect that enhances sorghum growth. Common PSMs include phosphate-solubilizing bacteria, e.g., *Bacillus*, *Pseudomonas*, *Burkholderia*, *Rhizobium*, and *Enterobacter*, and phosphate-solubilizing fungi, e.g., Aspergillus, Penicillium. These microorganisms work in two ways. They solubilize inorganic, mineral-bound P by secreting low-molecular-weight organic acids like gluconic, citric, oxalic, and malic acids. These organic acids decrease the rhizosphere pH and complex the Ca^2+^, Fe^3+^, and Al^3+^ ions that bind phosphate, thereby releasing inorganic phosphate in more available forms for uptake by the roots. Second, through the production of extracellular enzymes (phosphatases and phytases) that break down organic P compounds such as phytate to orthophosphate, PSMs aid in mineralizing organic P. The released phosphate is primarily H_2_PO_4_^−^ and HPO_4_^2−^ and can then be taken up by the phosphate transporters in the root cells of plants. PSMs can enhance sorghum root growth and stress tolerance via several plant growth-promoting abilities in addition to direct phosphorus solubilization. Many strains can produce phytohormones like indole-3-acetic acid (IAA), gibberellins, or cytokinins, which can help promote lateral root formation and root hair growth, thereby creating more surface area to take up nutrients. Some PSMs can also synthesize 1-aminocyclopropane-1-carboxylic acid (ACC) deaminase activity that can reduce the accumulation of stress-induced ethylene and sustain root growth in conditions of drought, salinity, and nutrient deficiency. Others synthesize siderophores, which aid in the acquisition of Fe and can also restrict the growth of pathogens by competing for available Fe in the rhizosphere [93]. Another source of resistance to soil-borne phytopathogens, which could be attributed to PSMs, is the production of lytic enzymes, antibiotics, volatile compounds, or hydrogen cyanide, but the latter is highly strain-specific. Moreover, PSM has been found to enhance resistance against soil-borne phytopathogens by producing siderophores, lytic enzymes, and hydrogen cyanide (HCN), which act as inhibitors of phytopathogens and plant health [94]. The integrated mechanisms result in improved nutrient assimilation, root growth, and stress resistance, ultimately contributing to sorghum productivity and sustainability in low-fertility soils [47,95,96]. But there is still limited evidence available for PSM strains specific to sorghum and their association with phosphate transporter regulation. Future research should investigate the interactions of PSMs with other nutrient-foraging microbes, such as diazotrophs, potassium-solubilizing bacteria, zinc-solubilizing microbes, and arbuscular mycorrhizal fungi to assess their ability to control N, P, and K uptake under low nutrient soils.

### 5.3. Mycorrhizal Fungi

Mycorrhizal fungi are symbiotic fungi that colonize plant root cortical tissues and develop extraradical hyphae that extend beyond the root surface and nutrient-depletion zone. These hyphae increase the effective absorptive area of the root system and can interact with other beneficial rhizosphere or hyphosphere microorganisms, such as phosphate-solubilizing bacteria and diazotrophs, thereby supporting nutrient mobilization and uptake. In sorghum, AMF colonization has been reported to enhance the uptake of N, P, S, Mg, Cu, and Zn, known to be involved in energy transfers and root development [97]. Moreover, AMF also induces resistance against drought by improving osmotic regulation, maintaining turgidity of the cells, and reducing transpiration(ref). Nitrogen-fixing bacteria, traditionally known as legumes, have an important role in sorghum, a cereal crop. *Rhizobium* and *Bradyrhizobium* species are examples that form loose associations with sorghum roots and can fix atmospheric N under low N conditions [98]. Mycorrhizal fungi and nitrogen-fixing bacteria may support sorghum nutrient acquisition through complementary mechanisms. Arbuscular mycorrhizal fungi extend extraradical hyphae beyond the root depletion zone, increasing access to relatively immobile nutrients such as phosphorus and contributing to nitrogen acquisition. AMF can absorb inorganic N forms, such as NH_4_^+^ and NO_3_^−^, and may assimilate nitrogen into amino acids such as arginine for transport through fungal hyphae. Near the arbuscule–root interface, nitrogen can be released and taken up by the host root through plant ammonium transporters. At the same time, diazotrophic bacteria can biologically fix atmospheric N_2_ and enrich the rhizosphere or endosphere nitrogen pool. Therefore, the combined activity of AMF and diazotrophic bacteria may improve sorghum nutrition by expanding the soil volume explored, increasing P and N mobilization, and supporting root-associated nutrient transfer [99,100].

### 5.4. Stress Tolerance Features of Endophytes

Endophytes are microorganisms (fungi and bacteria) that live in plants without causing any harm. These partners are the ones that are now gaining fame as allies to plants in distress. Under drought conditions, ethylene is a stress hormone that inhibits the elongation of the roots. Endophytes can help to keep ethylene to a minimum and promote the growth of the roots and water absorption in long dry spells. In sorghum, several bacterial root endophytes, including *Ochrobactrum* sp. EB-165, *Microbacterium* sp. EB-65, *Enterobacter* sp. EB-14, and *Enterobacter cloacae* EB-48, have been reported to promote growth and improve drought-stress tolerance. These effects are associated with plant-growth-promoting traits such as 1-aminocyclopropane-1-carboxylate (ACC) deaminase activity, phytohormone production, nutrient mobilization, and improved root development [101,102]. Fungal endophytes may also contribute to stress resilience; for example, the dark septate endophyte *Exophiala pisciphila* GM25 improved drought tolerance in sorghum by enhancing growth, dry matter accumulation, photosynthetic performance, and stress-related physiological responses [93,103]. While a few species of Fusarium are well-established pathogens of plants, their role in ecology may be strain- and host-specific. In the case of *Fusarium solani*, it is a reported cause of sorghum damping-off disease and should not be considered a general benefit to sorghum as an endophyte. Non-pathogenic strains (e.g., *F. solani* strain K in tomato) may be endophytic and modulate host defense mechanisms [104,105]. Plants under drought, heat, or pathogen-induced stress pathways produce ROS as part of normal stress signaling pathways, and excess production of ROS can cause damage to cellular membranes, proteins, and nucleic acids. Beneficial endophytes may enhance the ability of plants to respond to stress instead of removing ROS completely by increasing the antioxidant defense systems, especially enzymes like superoxide dismutase, catalase, peroxidase, and ascorbate peroxidase, which help to decrease oxidative injury while maintaining ROS for stress signaling [106,107]. The importance of understanding and harnessing the interaction between the microbes and the sorghum plant is underscored by the role of PGPMs, mycorrhizal fungi, N_2_-fixing bacteria, and endophytes in the resilience of sorghum.

## 6. Conclusions

The review demonstrates that the nutrient acquisition of sorghum is a highly complex process that is mediated by the rhizosphere microbiome assembly, plant transporter networks, and plant–microbe signaling. The roots of sorghum do not absorb the nutrients of the soil alone. They also determine the biological and chemical environment in their surroundings through the structure of the roots and exudations. The respective microbiome, in turn, influences nutrient availability, root development, and control of nutrient transport processes. Due to this, the molecular mechanism of nutrient uptake in sorghum should be perceived more as a joint action of plant and microbial action and not as a single plant characteristic. The review further points out that the absorption of N, P, and K is determined by different yet interrelated systems of transporters. Nitrogen uptake embraces nitrate and ammonium transporters; phosphorus uptake is mainly regulated by phosphate transporter families, and potassium uptake is regulated by both transporters and ion channels. Such systems also get affected by the presence of advantageous microbes like diazotrophs, phosphate-solubilizing microbes, potassium-mobilizing microorganisms, and arbuscular mycorrhizal fungi. Mycorrhizal symbiosis is one of such interactions that offers some of the best evidence that microbial partners might be able to directly facilitate nutrient movement and mediate processes related to transporters in plants. Nutrient transport is also connected to carbon status, hormone signaling, as well as stress responses. This implies that the enhancement of the nutrient use efficiency of sorghum will need to be done at the systems level. It may not be sufficient to concentrate on one transporter or one group of microbes. Rather, future strategies to improve nutrient use efficiency are to be based on the crosstalk between nutrient signaling pathways and the overall regulation of root–microbe interactions. Application-wise, this subject is very useful in sustainable agriculture. Increased knowledge of root–microbe control of transporter genes can be useful in the production of sorghum varieties that are better in nutrient use efficiency in low-input conditions. In general, root–microbe relationships are an important constituent of nutrient uptake in sorghum. Better knowledge of these interactions will be used to explain how sorghum can retain nutrient uptake during periods of stress and low fertility. It will also give a sounder scientific foundation for how to enhance the efficiency of nutrients, decrease reliance on fertilizer, and come up with more sturdy sorghum production systems in the future.

## Figures and Tables

**Table 1:** Sorghum-associated microbes, specific metabolites/signals, mechanisms, and plant responses related to nutrient acquisition.

Microbes	Specific Metabolite or Signal	Type	Mechanism	Plant Responses	Ref.
*Pseudomonas* sp.	Indole-3-acetic acid (IAA)	Root-associated PGPR	Produces an auxin-like compound that promotes root development and nutrient foraging under low-nutrient soil.	Increased sorghum stem diameter, shoot height, shoot dry weight, and root dry weight under low-nutrient soil conditions.	[81]
*Bacillus subtilis*	IAA, gibberellic acid (GA), siderophores; phosphate- and zinc-solubilization traits	Sorghum rhizosphere PGPR	Promotes root growth through phytohormone production and improves nutrient availability through P/Zn solubilization and siderophore production.	Improved sorghum growth under controlled/pot conditions; proposed as a growth-promoting bio-inoculant.	[82]
*Serratia rubidaea*	Organic acids/protons associated with phosphate solubilization	Phosphate-solubilizing bacterium	Solubilizes phosphate from phosphate-rich substrates through acid production and enzymatic activity, increasing plant-available phosphorus.	Evaluated as a phosphate-solubilizing bacterial (PSB) inoculant for sorghum grown in phosphorus-deficient soil.	[83]
*Aspergillus terreus*	Organic acids and phosphatase-related activity	Phosphate-solubilizing fungus	Releases fixed or sparingly soluble phosphate through acidification and enzymatic mineralization.	Improved P uptake and dry matter yield in sorghum when used with a phosphate source.	[84]
*Rhizophagus intraradices*	Extraradical hyphae; mycorrhizal nutrient-transfer interface	Arbuscular mycorrhizal fungus	Extends hyphae beyond the root depletion zone and transfers nutrients, especially P, through the symbiotic interface.	Evaluated with sorghum and associated with improved nutrient acquisition in phosphate-enriched substrates.	[83]
*Funneliformis mosseae*	Extraradical hyphae; mycorrhizal nutrient-transfer interface	Arbuscular mycorrhizal fungus	Increases soil exploration and supports nutrient uptake through the mycorrhizal pathway.	Evaluated as an AMF inoculant for sorghum growth and nutrient uptake.	[83]
Sorghum root exudate (sorgoleone)	Sorgoleone	Sorghum-specific root metabolite shaping microbial community structure	Alters bacterial and archaeal community structure and delays microbial network formation in the rhizosphere.	Influences sorghum rhizosphere assembly and can affect nitrification at specific stages.	[34]

## Data Availability

No new data were created or analyzed in this study. Data sharing is not applicable to this article.
